# Multi-omics analysis reveals interferon-stimulated gene OAS1 as a prognostic and immunological biomarker in pan-cancer

**DOI:** 10.3389/fimmu.2023.1249731

**Published:** 2023-10-20

**Authors:** Runyu Yang, Yue Du, Mengyao Zhang, Yi Liu, Hui Feng, Ruimin Liu, Bingyu Yang, Jiayi Xiao, Pengcheng He, Fan Niu

**Affiliations:** Department of Hematology, First Affiliated Hospital of Xi’an Jiaotong University, Xi’an, Shaanxi, China

**Keywords:** OAS1, tumor immune microenvironment, immunotherapeutic resistance, T-cell dysfunction, pan-cancer

## Abstract

**Introduction:**

OAS1(2’-5’-oligoadenylate synthetase 1) is a member of the Interferon-Stimulated Genes which plays an important role in the antiviral process. In recent years, the role of OAS1 in tumors has attracted attention, and it was found to be associated with prognosis in several tumors. However, the mechanism by which OAS1 affects tumors is unclear and pan-cancer study of OAS1 is necessary to better understand its implication in cancers.

**Methods:**

The expression, prognostic value, genetic alteration, alternative splicing events of OAS1 in pan-cancers were analyzed using TCGA, GTEx, HPA, GEPIA and OncoSplicing databases. OAS1 associated immune cell infiltration was evaluated using the ESTIMATE, xCell, CIBERSORT and QUANTISEQ algorithm. Single cell transcriptome data download using TISH database. Finally, the roles of the OAS1 on apoptosis, migration and invasion were investigated in two pancreatic cancer cells.

**Results:**

Our results revealed significant differences in OAS1 expression among various tumors, which had prognostic implications. In addition, we investigated the impact of OAS1 on genomic stability, methylation status, and other factors across different types of cancer, and the effects of these factors on prognosis. Notably, our study also demonstrated that OAS1 overexpression can contribute to CTL dysfunction and macrophage M2 polarization. In addition, cell experiments showed that the knockdown of OAS1 could reduce the invasive ability and increased the apoptosis rate of PAAD cells.

**Discussion:**

These results confirmed that OAS1 could be a prognostic biomarker and therapeutic target for its potential role in CTL dysfunction and macrophage M2 polarization.

## 1 Introduction

Cancer stands as one of the primary factors leading to mortality and imposes an increasing burden on public health (1, 2). While cancer treatment has made significant progress in recent years, particularly in immunotherapy (3, 4) such as immune checkpoint blockade (ICB) and chimeric antigen receptor T-cell therapy (CAR-T), patient response to immune checkpoint blockade remains disappointing (5–7), and the effectiveness of CAR-T therapy on solid tumors is still unsatisfactory (8). Currently, it is understood that T cell dysfunction is a significant factor contributing to resistance to immunotherapy (9, 10). This deactivation is closely linked with the intricate tumor microenvironment(TIME) (11). The infiltration of immunosuppressive cells and the dysfunction of effector cells within the tumor immune microenvironment collectively promote tumor initiation and progression. During the development and progression of tumors, various cell types could be recruited, including but not limited to T cells, macrophages, dendritic cells, neutrophils, B cells, and tumor-associated fibroblasts, together with extracellular matrix, forming the tumor immune microenvironment (12).T cells could lose their function in TIME, unable to effectively eliminate tumor cells, which is an important part of the development and progression of tumors (13–15). In recent years, other types of immune cells have received more attention. For example, it has been discovered that tumor-associated macrophages (TAM) usually exhibit M2 phenotype in the tumor immune microenvironment. TAM can promote tumor growth and contribute to tumor angiogenesis and tumor metastasis (16). The infiltration of myeloid-derived suppressor cells (MDSCs) has been found to affect tumor prognosis (17), tumor metastasis (18), and the activity of T cells and NK cells (19). Diverse cells within the tumor microenvironment can generate a plenty of cytokines via autocrine or paracrine secretion, constituting an intricate communication network, thereby further amplifying the complexity of the tumor microenvironment. Although cytokines like Interferon alpha (IFN-α) and Interferon gamma (IFN-γ) theoretically bolster the cytotoxicity of T cells against tumors (20), their therapeutic efficacy in clinical trials has been limited (21), indicating that cytokine function can also be dysregulated in the tumor microenvironment. Although insights into the TIME and the mechanisms of immune dysfunction have progressively expanded, our comprehension of these complex phenomena is still significantly restricted. The complex interplay of cells and molecules within the TIME, and their subsequent role in immune evasion and immunotherapeutic resistance call for more comprehensive investigations. Profound exploration of these mechanisms is crucial for the development of more effective immunotherapeutic strategies.

The OAS (2’-5’-oligoadenylate synthetase) gene family is a group of widely expressed genes in mammals that play an important role in the antiviral process (22). The gene family includes OAS1, OAS2, OAS3, and OASL. OAS1 is one of the most extensively studied members of the OAS family and plays an important role in regulating immune responses (23, 24). Cytokines such as IFN-α and IFN-γ can activate the Signal Transducer and Activator of Transcription 1 (STAT1), resulting in its phosphorylation (25). Subsequently, STAT1 forms a dimer with STAT2, which is capable of translocating into the nucleus to bind to the Interferon-Stimulated Response Element (ISRE) promoter. This leads to the upregulation of Interferon-Stimulated Genes (ISGs) (26), such as OAS1, which exhibit a significant increase in expression during viral, bacterial, and parasitic infections (27). OAS1 functions as a sensor of viral infections, recognizing dsRNA, a pathogen-associated molecular pattern (PAMP), and subsequently activating the innate immune system to exert antiviral activity. Previous studies also have shown that the polymorphism of the OAS1 gene is associated with susceptibility to various infectious diseases and disease severity (28). The polymorphisms rs10735079 (A) and rs6489867 (T) in the OAS1 gene are significantly associated with the severity of COVID-19 infection (29). The OAS1 gene generates multiple alternatively spliced variants that may affect the localization and function of the OAS1 protein (30). Variations in the alternative splicing forms of OAS1 have been reported as indicators of COVID-19 severity (31), with similar findings noted in other viral infections (32). Importantly, acquired mutations in OAS1 can lead to the patient’s autoinflammation and immunodeficiency. Mutant OAS1 proteins can exhibit antiviral activity even in the absence of dsRNA stimulation, which can result in alterations in the cellular transcriptome, accompanied by dysfunction in monocytes, macrophages, and B cells (33). Moreover, OAS1 has been reported to play a significant role in the development of numerous autoimmune diseases. There is a marked difference in the expression of OAS1 between patients with systemic lupus erythematosus (SLE) and healthy individuals, and it has been identified as a biomarker marker for SLE (34). Early increased OAS1 expression in rheumatoid arthritis is also linked to a poorer prognosis (35). In psoriasis, OAS1 can regulate the cell cycle and enhance the JAK1 signaling pathway (36), and it has been proposed as a diagnostic marker for dermatomyositis (37). The OAS1 variant (rs10774671) can predict the sensitivity of patients with multiple sclerosis to IFN-β treatment (38). Moreover, there is a significant increase in the expression of OAS1 in patients with type 1 diabetes, suggesting that the innate immune antiviral system may play a crucial role in the development and progression of type 1 diabetes (39). These studies indicate that OAS1 plays a crucial role in the immune response.

Current research suggests that OAS1 may be correlated with tumor prognosis in certain cancers. For instance, it has been discovered that OAS1 can influence the prognosis of patients by modulating inflammation and cell proliferation related pathways in gastric cancer (40). In breast cancer (41) and endometrial cancer (42), high expression of OAS1 is also associated with poor prognosis. In lung adenocarcinoma (43) and non-small cell lung cancer (44), there are studies reporting that OAS1 is related to the efficacy of immunotherapy and even traditional chemotherapy. Additionally, OAS1 can predict the efficacy of immunotherapy in bladder cancer patients, possibly because the expression of OAS1 is related to the infiltration of CD4^+^T cells, CD8^+^T cells, neutrophils, and dendritic cells in the tumor microenvironment of bladder cancer (43, 45).Our previous study revealed a significant association between OAS1 and the prognosis of pancreatic cancer (46). Furthermore, Lu et al. demonstrated that the overexpression of OAS1 in pancreatic cancer is related to cell apoptosis, the Notch signaling pathway, and the p53 signaling pathway (47). Moreover, overexpression of OAS1 in myeloid malignancies has been found to increase genomic instability, while polymorphism of the OAS1 gene, such as OAS1rs2660, is associated with increased susceptibility to prostate cancer (48). All these findings suggest that OAS1 may play a pivotal role in cancer development, especially in regulating the tumor immune microenvironment. However, despite progress in understanding the association of OAS1 with select cancers, no pan-cancer analysis has yet been performed to examine its potential use as a biomarker or therapeutic target. Therefore, more extensive pan-cancer studies are needed to gain comprehensive insights into the mechanisms through which OAS1 impacts tumor development and contributes to resistance against immunotherapy.

**Figure 1:**
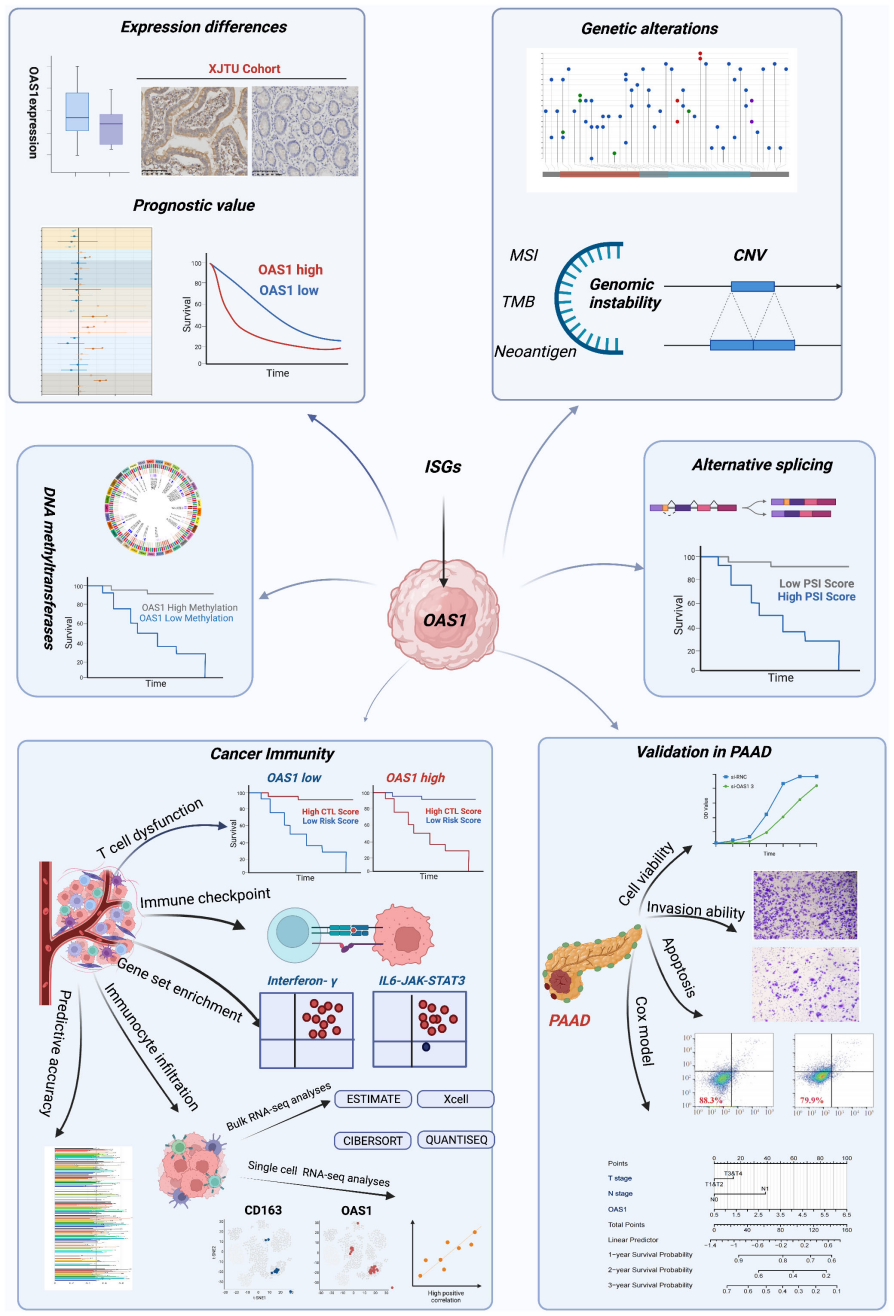
The workflow of this study. The workflow was created with BioRender.com.

In our study, we conducted a pan-cancer investigation of the OAS1 gene, employing bioinformatic analyses and integrating data from multiple databases (**Figure 1**). We found significant differences in OAS1 expression levels among various tumors, which had prognostic implications. To further clarify these findings, we verified them in pancreatic cancer. In addition, we investigated the impact of OAS1 on genomic stability, methylation status, and other factors across different types of cancer, and the effects of these factors on prognosis. Notably, our study also demonstrates that overexpression of OAS1 can induce CTL dysfunction through the IL6/JAK/STAT3 pathway, and it can also drive M2 macrophage polarization, highlighting its potential as a therapeutic target.

## 2 Materials and methods

### 2.1 Expression of OAS1 in tumors

The “Gene_DE” module in the Tumor Immune Estimation Resource (TIMER; http://timer.cistrome.org/) database (49) was accessed to compare the expression of OAS1 in different tumors. Gene transcriptome data in TPM format of normal samples from the Genotype-Tissue Expression (GTEx) project and pan-cancer samples from The Cancer Genome Atlas (TCGA) database were downloaded based on the UCSC Xena browser (https://xenabrowser.net/datapages/).The differences of OAS1 expression between tumor tissues and normal tissues were analyzed by wilcoxon rank sum test in R software, and differences in expression levels were visualized using ggplot2 (Version 3.4.2).The Clinical Proteomic Tumor Analysis Consortium (UPTAC, http://ualcan.path.uab.edu/analysis-prot.html) database, obtained from UALCAN (50) was utilized to compare differences in OAS1 protein expression among multiple tumor types at the proteomic level.The Gene Expression Profiling Interactive Analysis (GEPIA; http://gepia2.cancer-pku.cn/) database (46) was used to compare OAS1 expression in different tumor stages. Human Protein Atlas (HPA; www.proteinatlas.org) was a comprehensive human proteomics database that provides information on the expression and distribution of human proteins in different tissues, organs, and cell types. Tissue microarrays from HPA database showed differential expression of OAS1 in tumor tissue versus normal tissue by immunohistochemistry. Bubble maps of OAS1 related diseases were obtained from the Open Target Platform (https://platform.opentargets.org/) to identify potential disease associations with OAS1 expression.

### 2.2 Survival prognosis analysis

GEPIA is an online gene expression data analysis tool that can be used for survival analysis in different tumors. The “Survival Analysis” module was used to analyze the relationship between OAS1 expression and patient survival. median OAS1 expression values were selected as cut-off values and used to classify patients. the GEPIA database was used to obtain Kaplan–Meier plots for patient overall survival (OS) and disease-free survival (DFS). Hazard ratio (HR) was defined as the risk in the high OAS1 expression group divided by the risk in the low expression group, which was used to assess the effect of OAS1 expression on patient survival.

### 2.3 Analysis of OAS1 genetic alteration

To conduct gene mutation analysis, we downloaded the Simple Nucleotide Variation dataset of level 4 processed by MuTect2 software from the Genomic Data Commons (GDC; https://portal.gdc.cancer.gov/). The mutation data was integrated, and structural domain information for the proteins was obtained using the R package maftools (version 2.2.10). The Tumor Mutation Burden (TMB) was calculated using the “tmb”function of the R package maftools for each tumor sample. The TMB and gene expression data were then integrated, and a log2 (x+0.001) transformation was performed for each expression value to calculate the correlation between OAS1 expression and TMB scores. Microsatellite instability (MSI) scores for each tumor were obtained from a previous study (47). We integrated the MSI and gene expression data of the samples to calculate the correlation between OAS1 expression and MSI scores. Finally, neoantigen data for each tumor were obtained from a previous study (51). The neoantigen and gene expression data were integrated to calculate the correlation between OAS1 expression and immune neoantigen scores.

### 2.4 Analysis of OAS1 alternative splicing events

To identify clinically relevant alternative splicing (AS) events, we used the ClinicalAS module of the OncoSplicing website (http://www.oncosplicing.com/) (52). We searched for AS events of OAS1 that were included in both the SplAdder and SpliceSeq projects. We selected OAS1_alt_3prime_56568 for the further study. The PanDiff plots were presented to compare percent spliced-in(PSI)differences in AS events between cancers and GTEx normal tissues. For further analysis, we plotted Kaplan-Meier curves to investigate the prognostic significance of the OAS1_alt_3prime_56568 in pan-cancer.

### 2.5 OAS1 and tumor immune microenvironment

To assess stromal, immune, and overall tumor purity (ESTIMATE) scores for each patient in each tumor, we utilized the R package ESTIMATE (version 1.0.13) (53). Additionally, we employed the deconvo_tme function in the R package IOBR (version 0.99.9) (54) with the xCell (55) algorithm to re-evaluate major immune cell infiltration scores based on gene expression. To further evaluate the infiltration score of M2 subtype macrophages, we used both CIBERSORT (56) and QUANTISEQ (57) algorithms. Finally, we investigated the correlation between OAS1 expression and immune cell infiltration scores using Pearson correlation analysis to calculate the correlation coefficient between these two factors. We utilized the Tumor Immune Dysfunction and Exclusion (TIDE, http://tide.dfci.harvard.edu) database (58) to assess the prognostic significance of OAS1 expression and cytotoxic T lymphocyte (CTL) infiltration in patients. The T cell dysfunction score for each gene is determined by calculating the Z-score of the Wald test, which is obtained by dividing the coefficient by its standard error. The utilization of Z-Score standardization allows for the transformation of data with diverse metrics into a standardized metric, thus enhancing the comparability of the data. Additionally, we compared OAS1 with other indicators for predicting the efficacy of immunotherapy using the TIDE database. The Tracking Tumor Immunophenotype database (TIP, http://biocc.hrbmu.edu.cn/TIP/) (59) was used to evaluate the anticancer immune status in seven different stages of the tumor immune cycle, and the Gene Set Cancer Analysis database (GSCA, http://bioinfo.life.hust.edu.cn/GSCA/) was used to analyze the relationship between OAS1 and exhausted T cell scores (60).

### 2.6 Gene set enrichment analysis & protein-protein interaction network construction

The pan-cancer samples were segregated into two groups using the median expression of OAS1 across all samples as a threshold. We then compared the differences in gene expression between the two groups and ranked them based on the fold change values. We applied the Gene Set Enrichment Analysis (GSEA) method to study all samples by analyzing gene sets h.all.v7.4.symbols.gmt which were downloaded from the Molecular Signatures Database (http://www.gsea-msigdb.org/gsea/downloads.jsp) (61) using the R package clusterProfiler (version 4.0.5) (62). The primary statistic for examining gene set enrichment results was the normalized enrichment score (NES). Moreover, the false discovery rate (FDR) was estimated as the probability that a gene set with a given NES represents a false positive finding. The indicators of enrichment were NES and FDR, and we considered gene sets with |NES|>1 and FDR < 0.25 to be significantly enriched. Finally, we visualized the outcomes using ggplot2 (version 3.4.2) package. Based on the expression levels of OAS1, we divided the TCGA_PAAD cohort into high OAS1 expression group and low OAS1 expression group. The R package DESeq2(version 1.38.3) was used to analyze the differentially expressed genes (with criteria of |log2FC| > 1.5 and adj.pvalue < 0.05). The differentially expressed genes were then input into the STRING database (https://cn.string-db.org/) with a minimum required interaction score module set at 0.7 to obtain the PPI network graph. The R package igraph(version 1.5.0) was employed for constructing the network modules, while the clusterProfiler package was utilized for conducting enrichment analysis.

### 2.7 Expression level of OAS1 at the single−cell level

Tumor Immune Single-cell Hub (TISH, http://tisch.comp-genomics.org/home/) database (63) was used to download single-cell transcriptome data (**Table S4**). We utilized the R package Seurat (version 4.3.0) for data processing and analysis. The “Read10X_h5” function was used to read the data, while “CreateSeuratObject” was used to build the SeuratObject. Data normalization was performed using LogNormalize with a scale factor of 10000. Highly variable genes were selected using the “FindVariableFeatures” function with the parameter ‘nfeatures’ set as 2000.Principal component analysis (PCA) was performed on the dataset using the “RunPCA” function. The top 10 principal components were used to construct a k nearest neighbor graph using the “FindNeighbors” function. Finally, we reduced the dimensions of the data using the uniform manifold approximation and projection (UMAP) algorithm via the “RunUMAP” function for visualization purposes.To visualize the gene expression patterns within the dataset, we used the “FeaturePlot” function to display the co-expression relationships between two different genes. Additionally, we utilized the “getScatterplot” function to calculate and visualize the correlation between the expression of two different genes at the single-cell level.

### 2.8 IHC analysis of OAS1 in pancreas tumor samples and evaluation

Nine pancreatic cancer patients’ samples were selected in the pathology department of the First Affiliated Hospital of Xi ‘an Jiaotong University. All experiments on tumor samples from patients performed were under the supervision of the Ethics Committee of the First Affiliated Hospital of Xi’an Jiaotong University (XITU1AF2022LSK-339). They were all confirmed as pancreatic ductal carcinoma through tissue biopsy. Samples after surgical resection were fixed in formalin, embedded in paraffin and stored. Formalin-fixed, paraffin-embedded sections were cut into 4-μm sections. The sections were deparaffinized and rehydrated using xylene and graded alcohol solutions. They were blocked with 2% bovine serum albumin, incubated with specific primary antibodies for 12 hours at 4°C followed by incubation with a biotinylated secondary antibody. Subsequently, diaminobenzidine (DAB) was added dropwise over a 3 to 5 minutes period and sections were then counterstained with hematoxylin. The primary antibodies included rabbit monoclonal antibody of recombinant Anti-OAS1 (dilution 1:500, cat. No: ab232862, Abcam).

Immunohistochemical staining results were scored according to the following criteria. According to the cell staining intensity, the score is grade 4, zero for no positive staining (negative), one for light yellow (weak positive), two for tan (positive) and three for tan (strong positive). According to the percentage of positive cells, it is rated as grade 4, with ≤25% as 1 point, 25%-50% as 2 points, 51%-75% as 3 points, and > 75% as 4 points. The final score can be obtained by multiplying the two scores. 3 views of cancer tissues and 3 of para cancerous tissues were randomly selected for each sample by pathologist. And then all of views were scored by three independent researchers or pathologists in a double-blind condition.

### 2.9 Cell culture

Panc-1 and Bxpc-3 cells were maintained in Dulbecco’s modified Eagle medium (DMEM) containing 10% FBS in an incubation at 37°C, with 5% CO_2_. Petri dish size is 10 cm. The passage ratios of Panc-1 and Bxpc-3 were 1: 3 and 1: 4, respectively.

### 2.10 Transfection experiment

The cells were reseeded at a density of 5×10^4^ cells per well in a 6-well plate, 24 hours prior to transfection. Subsequently, the cells were incubated in a 5% CO2 incubator at 37°C.The cells were transfected when the cell density reached 70%–80%. The transfection complex was prepared: 4μL of each siRNA (10 μM) was added to 100μL of serum-free medium, and the mixture was allowed to stand for 5 minutes at room temperature. 12μL of Lipofectamine™ RNAiMAX (Invitrogen, the US) transfection reagent was added. Allow to stand at room temperature for 15 minutes. The transfection complex was uniformly added into the 6-well plate, and returned to the incubator for continuous culture for 6 h. The medium containing the transfection complex was discarded and washed once with 2 mL PBS. Then 2mL cell culture medium containing 10% serum was added and the culture was continued for 18h in a 5%CO_2_ incubator at 37°C.

### 2.11 Knockdown evaluation by RT-PCR

The cells were collected 24h after transfection. Total RNA was extracted using RNA extraction kit following rigorously the manufacture’s instruction. Then prepare a reaction mixture before reverse transcription: gDNA remover 1.0ul, 10×gDNA remover Buffer 1.0ul, Total RNA 1000ng, RNase Free dH_2_O up to 10.0. Incubate the mixture for 2 min at 42°C, 5 min at 60°C and cool rapidly on ice. Add the following reagents 10.0 ul to give a final total of 20 μl: 5×Goldenstar™ Buffer 4.0 ul, dNTP Mix 1.0 ul, Goldenstar™ Oligo (dT)17 1.0 ul, Randomer 1.0 ul, DTT 1.0 ul, Goldenstar™ RT6 1.0 ul, RNase Free ddH_2_O up to 20.0 ul. The reverse transcription reaction conditions were as follows: 25°C for 10 minutes, 55°C for 50 minutes, 85°C for 5 minutes and 4°C for +∞. Reverse transcription polymerase chain reaction (RT-PCR) primer sequences in **Supplement Table 1**. RT-PCR reaction system was as follows (formulated on ice):2 ×T5 Fast qPCR Mix 10.0 ul, 10 μM Primer F 0.8 ul, 10 μM Primer R 0.8 ul, 50 ×ROX Reference Dye II 0.4 ul, Template DNA 0.5 ul, ddH_2_O up to 20.0 ul. The two-step quantitative polymerase chain reaction procedure included Holding stage and Cycling Stage. the former keeps 95°C for 30 s. The latter include 95°C for 5 s, 55°C for 30 s and 72°C for 30 s, 40 cycles total.All the above reagents are from Tsingke Biotechnology Co.,Ltd.

### 2.12 Cell viability assay by CCK-8

Bxpc-3 and Panc-1 cells were seeded at a density of 3000 cells per well in 96-well plates and cultured in an incubator at 37°C for about 4 hours to attachment, followed by treatment with siRNA for 24 hours. After 24h, the 96-well plate was taken out and the medium were replaced by DMEM 10% FBS containing 10% CCK8 (Bimake). After 2 hours of incubation, the absorbance at 450nm was determined using a micro-UV-visible spectrophotometer (NanoDrop One/One C Thermo, USA). Cell viability was calculated strictly following the instructions.

### 2.13 Invasion assay

Cell migration chambers consisting of 24 well plates were used for invasion assays (Corning, USA). Transwell plates were coated with Matrigel^®^ (diluted with serum-free DMEM 1:5; Thermo Fisher Scientific) and 500 μL of DMEM containing 20% FBS was added to the lower well. Cells were transfected by siRNA and incubated for 24 hours. Then a total of 5 × 10^4^ knocked down or not cells were added to the Matrigel^®^-coated chamber without FBS. After 48 hours of culture, the cells transferred to the other side were fixed with 4% paraformaldehyde for 20 minutes, washed 3 times with PBS, and stained with 0.1% crystal violet for 20 minutes. The migrated cells were photographed with a microscope and camera and then counted in three different areas.

### 2.14 Apoptosis detected by flow cytometry

The cells were cultured at a density of 5×10^4^ cells per well in 6-well plates. After their adhesion, cells were transfected by siRNA and incubated for 24 hours. Cells were collected using trypsin (Gibco) and centrifuged at 1000g for 5 minutes. Apoptosis was assessed using apoptosis assay (Biolegend). Briefly, after the cells were re-suspended with 100 μl binding solution, 5 μl AnnexinV-APC and 10 μl PI were added and mixed. After an incubation protected from light at room temperature (20–25 °C) in the dark for 15 minutes, 400 μl of binding solution was added to each sample. Flow cytometry (CantoII, BD, the US) tests were performed immediately and data were treated and plotted by FlowJo (version 10).

### 2.15 Statistical analyses

All bioinformatics analysis were conducted through R software (version 4.2.2). The above visualization was performed with R software and GraphPad Prism 9. The comparison of difference between two groups was analyzed using Wilcoxon rank-sum test. The comparison of difference between three groups or more groups was analyzed using the Kruskal–Wallis test. The paired t-test was used to study the differences in OAS1 expression between pancreatic cancer tissues and adjacent normal tissues in the GSE28735 dataset. The log-rank test was used to analyze patient survival significance. Pearson’s or Spearman’s correlation coefficients were used to quantify the correlations. p-Values less than 0.05 (p < 0.05) were considered significant.

## 3 Results

### 3.1 The expression of OAS1 in different cancers

**Figure 2:**
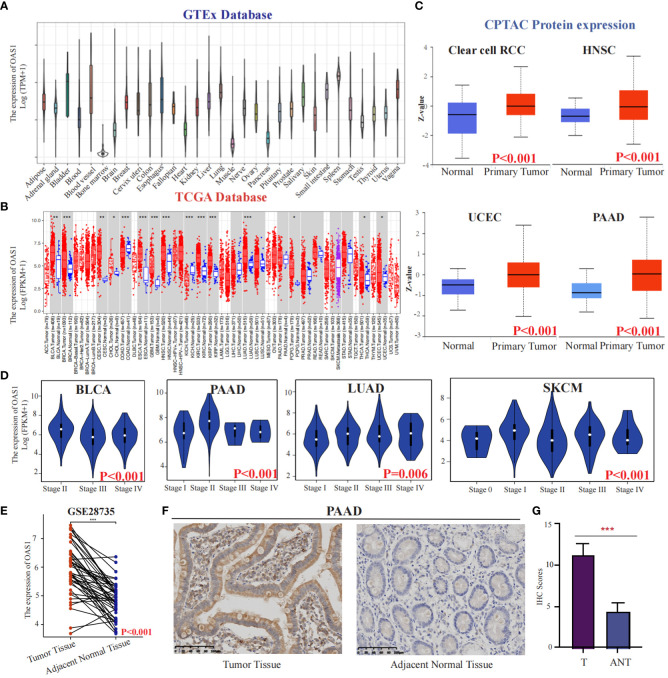
The expression of OAS1 in different cancers. The differences in OAS1 expression levels among various normal tissues can be found in the GTEx database**(A)**.The expression of OAS1 was significantly overexpressed in BLCA, BRCA, CESC, CHOL, COAD, ESCA, GBM, HNSC, KIRC, KIRP, LUAD, PCPG, THCA, and UCEC in TCGA database **(B)**. The OAS1 protein was significantly overexpressed in Clear cell RCC, HNSC, UCEC, and PAAD in the CPTAC database **(C)**. There was a correlation between OAS1 expression and the pathological stage of the tumor in BLCA, PAAD, LUAD, and SKCM **(D)**. The boxplot illustrates the differential expression of OAS1 between pancreatic cancer tissues and adjacent non-cancerous tissues in the GSE28735 dataset **(E)**. The OAS1 protein was significantly overexpressed in PAAD using immunohistochemistry data from the XJTU cohort **(F, G)**. (*p<0.05, **p<0.01, ***p<0.001).

We initially investigated the expression of OAS1 in the CCLE database and observed high expression levels in organs of the digestive system such as the pancreas and intestine cancer cells (**Figure S1A**). In addition, exploring diseases associated with OAS1 using OpenTargets revealed that it is primarily linked to COVID-19 and chronic lymphocytic leukemia (**Figure S1B**). To better understand the role of OAS1 in cancer, we analyzed its expression changes in The Cancer Genome Atlas (TCGA) database, which contains descriptions of 33 types of cancer (**Table S3**). Through analysis of the GTEx database, we found that OAS1 exhibits higher expression levels in bladder tissues (**Figure 2A**). Furthermore, our analysis revealed significant overexpression of OAS1 mRNA in several tumor tissues, including BLCA, BRCA, CESC, CHOL, COAD, ESCA, GBM, HNSC, KIRC, KIRP, LUAD, PCPG, THCA, and UCEC (**Figure 2B**). Moreover, we examined OAS1 protein expression through analysis of the CPTAC database and discovered significant increases in Clear cell RCC, HNSC, UCEC, and PAAD (**Figure 2C**). Additionally, we evaluated the correlation between OAS1 expression and tumor pathological stage and found significant associations in BLCA, PAAD, LUAD, and SKCM (**Figure 2D**). It is worth noting that in the GSE28735 dataset, we analyzed the expression levels of OAS1 in pancreatic cancer tissues and adjacent normal tissues from 45 patients. The results indicated a significantly higher expression of OAS1 in pancreatic cancer tissues compared to adjacent normal tissues (**Figure 2E**). To confirm these results, we performed immunohistochemistry using a XJTU PAAD cohort. Results demonstrated expression levels of OAS1 protein greatly increased in PAAD (**Figures 2F, G**). Finally, we examined immunohistochemical data from the HPA database and observed significantly elevated expression levels of OAS1 in LUAD, STAD, PAAD, KIRC, PRAD, and OV (**Figure S1C**). Overall, our results suggest that OAS1 may play an oncogenic role in various tumors, and its clinical application value is worth exploring at the pan-cancer level.

### 3.2 The prognostic value of OAS1 expression

**Figure 3:**
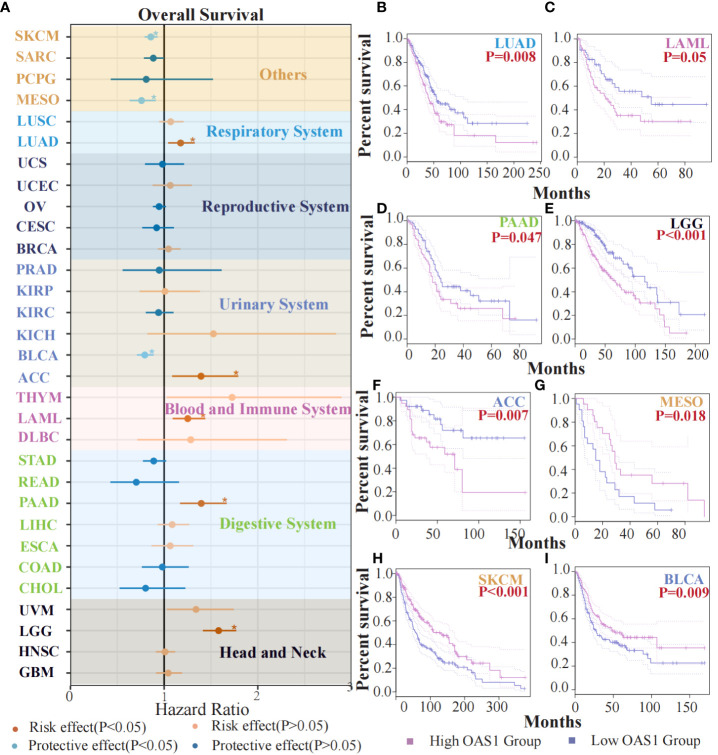
The prognostic value of OAS1 expression. The OAS1 expression was associated with the prognosis of several tumor types **(A)**. The higher OAS1 expression were linked to shorter OS in LUAD (HR=1.32, p=0.008), LAML (HR=1.43, p=0.05), PAAD (HR=1.66, p=0.047), LGG (HR=1.76, p<0.001), ACC (HR=1.79, p=0.007) **(B-F)**. The higher OAS1 expression were linked to longer OS in MESO (HR=0.91, p = 0.018), SKCM (HR=0.93, p < 0.001), BLCA (HR=0.88, p = 0.009) **(G-I)**. (*p<0.05).

To evaluate the potential prognostic significance of OAS1 in various human tumors, we analyzed its correlation with overall survival (OS) using data from the TCGA database. Forest plot revealed that OAS1 expression was associated with the prognosis of several tumor types (**Figure 3A**). Specifically, we observed that high OAS1 expression levels were linked to shorter OS compared to low OAS1 expression levels in LUAD (HR=1.32, p=0.008), LAML (HR=1.43, p=0.05), PAAD (HR=1.66, p=0.047), LGG (HR=1.76, p<0.001), ACC (HR=1.79, p=0.007) (**Figures 3B-F**), and in MESO (HR=0.91,p = 0.018), SKCM (HR=0.93, p < 0.001), BLCA (HR=0.88,p = 0.009) (**Figures 3G-I**), the opposite results were observed. Furthermore, we found that higher expression of OAS1 was linked to shorter disease-free survival (DFS) among patients with LUAD (HR=1.4, p =0.019), LGG (HR=1.6, p =0.005), PRAD (HR=1.6, p =0.028), UVM (HR=3.4, p =0.009) (**Figures S2B-E**). Our findings highlight the crucial role of OAS1 as a novel biomarker for predicting the prognosis of patients with various tumors.

### 3.3 The role of OAS1 in PAAD

**Figure 4:**
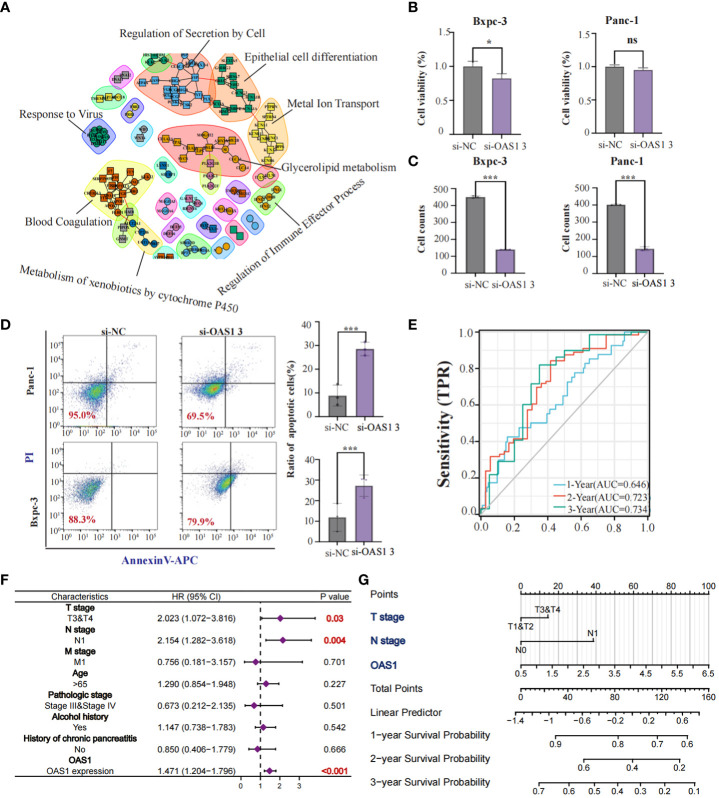
The role of OAS1 in PAAD. The PPI network plots showed that differential gene interactions were associated with epithelial differentiation **(A)**. The significant reduction of cell viability **(B)** and invasion capability **(C)** was observed in OAS1 knockdown cell line Bxpc-3 and Panc-1. More apoptosis was observed in OAS1 knockdown cell line Bxpc-3 and Panc-1 **(D)**. The expression of OAS1 in pancreatic cancer can predict overall survival at 1 year (AUC=0.646), 2 years (AUC=0.723) or 3 years (AUC=0.734) **(E)**. A survival prediction model for pancreatic cancer patients was created based on expression of OAS1 and pathological staging using the Cox model and visualized it using Nomogram plots **(F, G)**. ns, no significance, *p<0.05, ***p<0.001.

Our study revealed that OAS1 is highly expressed in tumor tissues compared to normal tissues and is associated with the prognosis of patients with various types of tumors, including pancreatic cancer. We aimed to further explore the role played by OAS1 in pancreatic cancer development. Initially, we analyzed differential genes between patients in the OAS1 high expression group and those in the low expression group and conducted Protein-protein interaction analysis. Our PPI network plots showed that differential gene interactions were associated with epithelial differentiation, which has been linked to malignant tumor progression (**Figure 4A**). Subsequently, To investigate the implication of OAS1 in tumor cell progression, we performed the knock-down test using siRNAs in two pancreatic cancer cell lines. Three siRNA were designed and their knock-down efficiency verified using RT-PCR. Results indicated that siRNA3 represented the best knockdown efficiency (up to 70%) (**Figure S2A**), making it the ideal choice for further research. To evaluate the impact of OAS1 on the invasion ability of pancreas cancer, trans-well assays were performed using cells Bxpc-3 and Panc-1after OAS1 knock-down. Our results demonstrated that in both Bxpc-3 (siRNC: 402 ± 3.05, siRNA3: 145± 11.67) and Panc-1 (siRNC: 450 ± 6.51, siRNA3: 140± 1.00) cell lines, the invasion ability of pancreatic cancer cells was significantly reduced after OAS1 knockdown (**Figure 4C**, **Figure S2B**). Additionally, to evaluate its involvement in apoptosis, apoptosis assay by flow cytometry was performed, and showed that knockdown of OAS1 increased apoptosis in both Bxpc-3 (siRNC: 11.93 ± 6.77, siRNA3: 27.29 ± 5.20) and Panc-1 (siRNC: 12.72± 1.01, siRNA3: 21.97± 3.34) cell lines (**Figure 4D**). However, cell viability tests after knocking down OAS1 showed that it had a moderate effect (less than 50%) on the viability of Bxpc-3 (siRNC: 1± 0.07, siRNA3: 0.82± 0.06) and did not affect the viability of Panc-1 cells (siRNC: 1± 0.03, siRNA3: 0.95± 0.03) (**Figure 4B**).

Furthermore, AUC plots showed that the expression of OAS1 in patients with pancreatic cancer can predict overall survival at 1 year (AUC=0.646), 2 years (AUC=0.723) or 3 years (AUC=0.734) (**Figure 4E**). Based on the expression of OAS1 and pathological staging, we developed a survival prediction model for pancreatic cancer patients using the Cox model and visualized it using Nomogram plots (**Figures 4F, G**). In conclusion, our findings suggest that OAS1 could be a potential biomarker for pancreatic cancer prognosis and a target for therapeutic intervention.

### 3.4 Genetic alterations and prognostic implications of OAS1 gene in tumors

**Figure 5:**
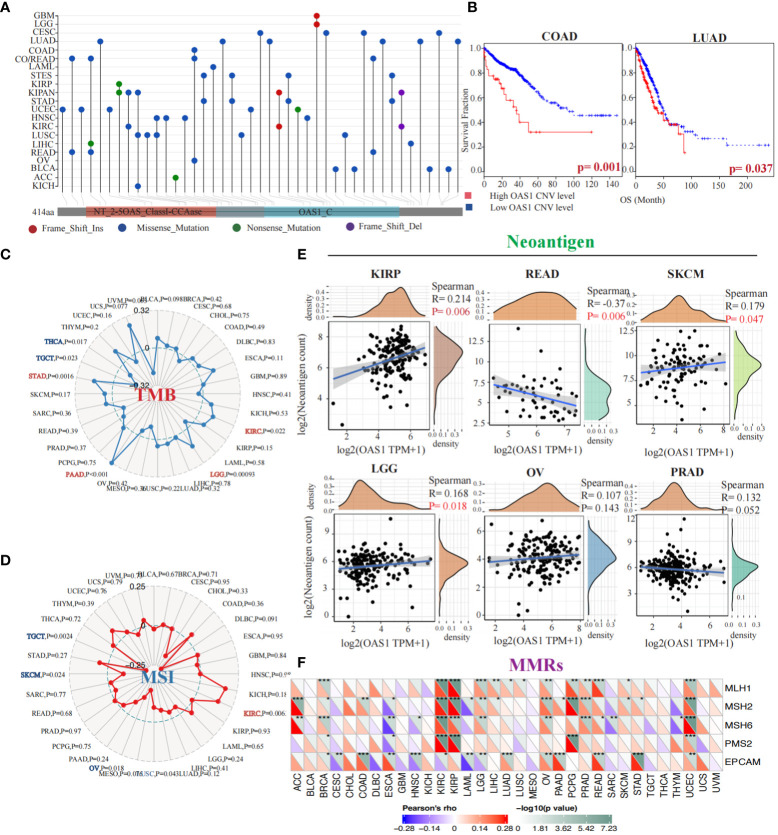
Genetic Alterations and Prognostic Implications of OAS1 Gene in Tumors. The OAS1 gene mutation status in various tumor types **(A)**. The high OAS1 CNV levels were associated with shorter survival rates in COAD (HR=2.24, p = 0.001) and LUAD (HR=1.92, p=0.037) **(B)**. The OAS1 expression was positively correlated with TMB in four cancers (KIRC, LGG, PAAD, STAD) **(C)** and with MSI only in KIRC **(D)**. The expression level of OAS1 was positively correlated with the score of neoantigen in KIRP, SKCM, LGG, OV, PRAD, and negatively correlated with READ **(E)**. The OAS1 expression was related to MMR systems, such as MLH1, MSH2, MSH6, PMS2, and EPCAM **(F)**. (*p<0.05, **p<0.01, ***p<0.001).

To evaluate OAS1 genetic alteration in cancers, we conducted a comprehensive analysis of the OAS1 gene mutation status in various tumor types. Our findings indicate that different tumors exhibit distinct genetic alterations of the OAS1 gene, including frameshift insertion, missense mutation, nonsense mutation, and frameshift deletion (**Figure 5A**). Furthermore, we investigated the impact of copy number variations (CNVs) in the OAS1 gene on the prognosis of cancer patients. We stratified cancer patients based on their OAS1 CNV levels and found that high OAS1 CNV levels were associated with shorter survival rates in COAD (HR=2.24, p = 0.001) and LUAD (HR=1.92, p=0.037) (**Figure 5B**). To assess the relationship between OAS1 expression and other genomic alterations commonly observed in cancer that impact patient prognosis and treatment response, we compared TMB, MSI, and neoantigens at the pan-cancer level. Radar plots revealed that OAS1 expression was positively correlated with TMB in four cancers (KIRC, LGG, PAAD, STAD) and with MSI only in KIRC. By contrast, it was negatively correlated with TMB in two cancers (THCA, TGCT) and with MSI in three cancers (TGCT, SKCM, OV) (**Figures 5C, D**). We also examined the effect of OAS1 expression on the formation of neoantigens in different tumors. Results showed that the expression level of OAS1 was positively correlated with the score of neoantigen in KIRP, SKCM, LGG, OV, PRAD, and negatively correlated with READ (**Figure 5E**). Finally, we explored the impact of OAS1 gene expression on the Mismatch Repair System (MMRs) in tumors. MMRs play a critical role in maintaining DNA replication accuracy and integrity. Our analysis revealed a significant association between OAS1 expression in various tumors and the expression of genes related to MMR systems, such as MLH1, MSH2, MSH6, PMS2, and EPCAM (**Figure 5F**).

### 3.5 OAS1 gene methylation and alternative splicing events in tumorigenesis and prognosis

**Figure 6:**
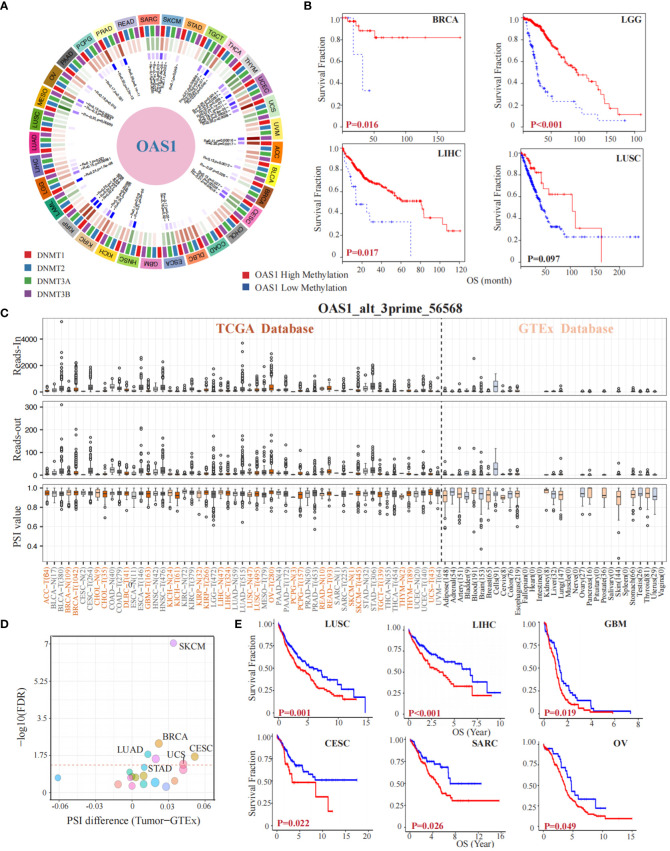
OAS1 Gene Methylation and Alternative Splicing Events in Tumorigenesis and Prognosis. The OAS1 gene is associated with DNA methyltransferases in multiple types of tumors **(A)**. The higher levels of OAS1 promoter methylation were associated with better prognosis in BCRA (p = 0.016), LGG (p < 0.001), LIHC (p = 0.017), and LUSC (p = 0.097) **(B)**. A substantial correlation was observed between tumorigenesis and the alternative splicing variant OAS1_alt_3prime_56568 **(C)**, especially in LUAD, STAD, UCS, BRCA, CESC, and SKCM **(D)**. High PSI values were linked to poorer prognosis in LUSC (p = 0.001), LIHC (p < 0.001), GBM (p = 0.019), CESC (p = 0.022), SARC (p = 0.026), and OV (p = 0.049) **(E)**.

To investigate the implication of OAS1 in epigenetics, we preformed a gene methylation association evaluation. Our study revealed that the OAS1 gene is associated with DNA methyltransferases in multiple types of tumors (**Figure 6A**). Furthermore, we analyzed the impact of OAS1 promoter methylation on prognosis and found that higher levels of OAS1 promoter methylation were associated with better prognosis in tumor types such as BCRA (p = 0.016), LGG (p < 0.001), LIHC (p = 0.017), and LUSC (p = 0.097) (**Figure 6B**). We also investigated the alternative splicing events of OAS1 gene and discovered a significant correlation between tumorigenesis and OAS1_alt_3prime_56568 (**Figure 6C**) in various cancers such as LUAD, STAD, UCS, BRCA, CESC, and SKCM (**Figure 6D**). Finally, we found that this specific alternative splicing event was associated with prognosis in patients with multiple tumors: high PSI values were linked to poorer prognosis in LUSC (p = 0.001), LIHC (p < 0.001), GBM (p = 0.019), CESC (p = 0.022), SARC (p = 0.026), and OV (p = 0.049) (**Figure 6E**). In summary, our findings suggest that OAS1 promoter methylation and alternative splicing events may have important implications for diagnosis, prognosis, and treatment of different types of tumors.

### 3.6 OAS1 impacted CTL function and contributed to increased resistance to immunotherapy

**Figure 7:**
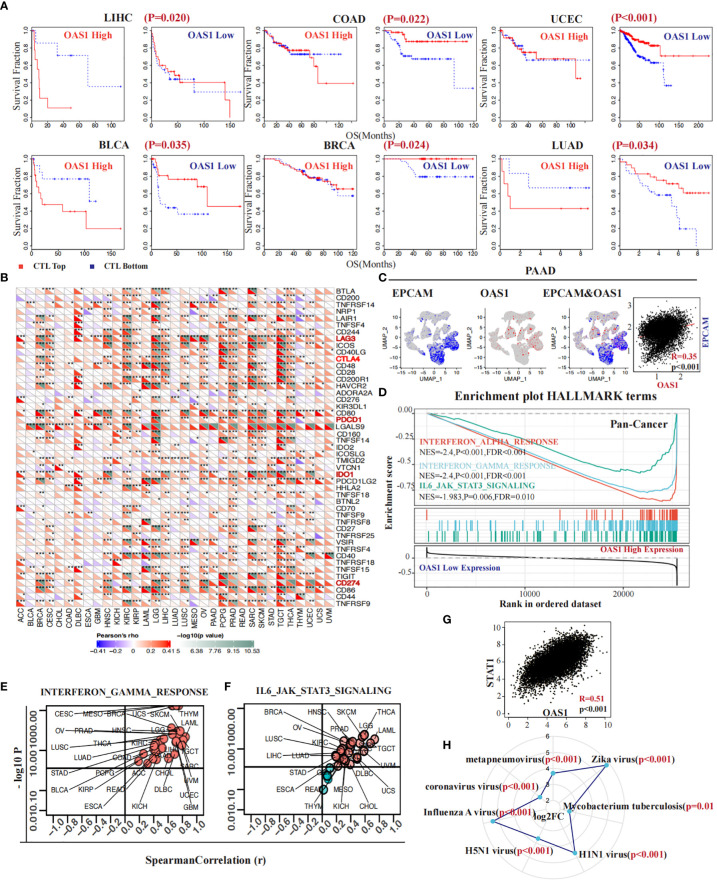
OAS1 impacted CTL function and contributed to increased resistance to immunotherapy. The high expression of OAS1 diminishes or even reverses the benefits of CTL infiltration levels on patient survival in LIHC (p = 0.020), COAD (p = 0.022), UCEC (p < 0.001), BLCA (p = 0.035), BRCA (p = 0.024), and LUAD (p = 0.034) **(A)**. In multiple tumors, overexpression of OAS1 corresponds with overexpression of immune checkpoint markers, such as LAG3, CTLA4, PDCD1 (PD-1), IDO1, and CD274 (PD-L1) **(B)**. OAS1 expression coincided with EPCAM at the single-cell level in PAAD **(C)**. Overexpression of OAS1 is linked to the activation of the interferon α (NES = -2.4, FDR <0.001) **(D)** and γ signaling pathways (NES = -2.4, FDR <0.001) **(D, E)**, as well as the IL6-JAK-STAT3 signaling pathway (NES =-1.98, FDR = 0.01) **(D, F)**. Infection by certain pathogens significantly increases OAS1 expression **(H)**. (*p<0.05, **p<0.01, ***p<0.001).

The infiltration of cytotoxic T lymphocytes is significantly associated with response to immunotherapy and tumor prognosis. As previously identified, greater infiltration of these cells when the OAS1 gene is expressed at low levels indicates a better prognosis. However, this situation changes dramatically when OAS1 expression is higher. In several cancers, such as LIHC (p = 0.020), COAD (p = 0.022), UCEC (p < 0.001), BLCA (p = 0.035), BRCA (p = 0.024), and LUAD (p = 0.034) (**Figure 7A**), high expression of OAS1 diminishes or even reverses the benefits of CTL infiltration levels on patient survival. Further investigation revealed that OAS1 can upregulate the expression of immune checkpoint markers associated with T cell dysfunction, including LAG3, CTLA4, PDCD1 (PD-1), IDO1, and CD274 (PD-L1) (**Figure 7B**). At the single-cell level in PAAD, we observed that OAS1 gene expression coincides with EPCAM, a label of malignant cells (R = 0.35, p < 0.001) (**Figure 7C**). Pan-cancer gene set enrichment analysis showed that high expression of OAS1 is linked to the activation of multiple signaling pathways, such as the interferon α (NES = -2.4, FDR <0.001) and γ signaling pathways (NES = -2.4, FDR <0.001), as well as the IL6-JAK-STAT3 signaling pathway (NES =-1.98, FDR = 0.01) (**Figure 7D**). Furthermore, almost all cancers demonstrated a positive correlation between OAS1 expression and GSVA scores of the interferon-γ signaling pathway (**Figure 7E**) and IL6-JAK-STAT3 signaling pathway (**Figure 7F**). We also found a positive correlation between the expression of OAS1 and STAT1 (R = 0.51, p < 0.001) (**Figure 7G**). Finally, we discovered that infection by certain pathogens significantly increases OAS1 expression, especially mycobacterium tuberculosis (log FC = 2.6, p = 0.01) and Zika virus (log FC = 5.8, p < 0.001) (**Figure 7H**), which were prone to immune escape.

### 3.7 Correlations between OAS1 expression and tumor immune microenvironment

**Figure 8:**
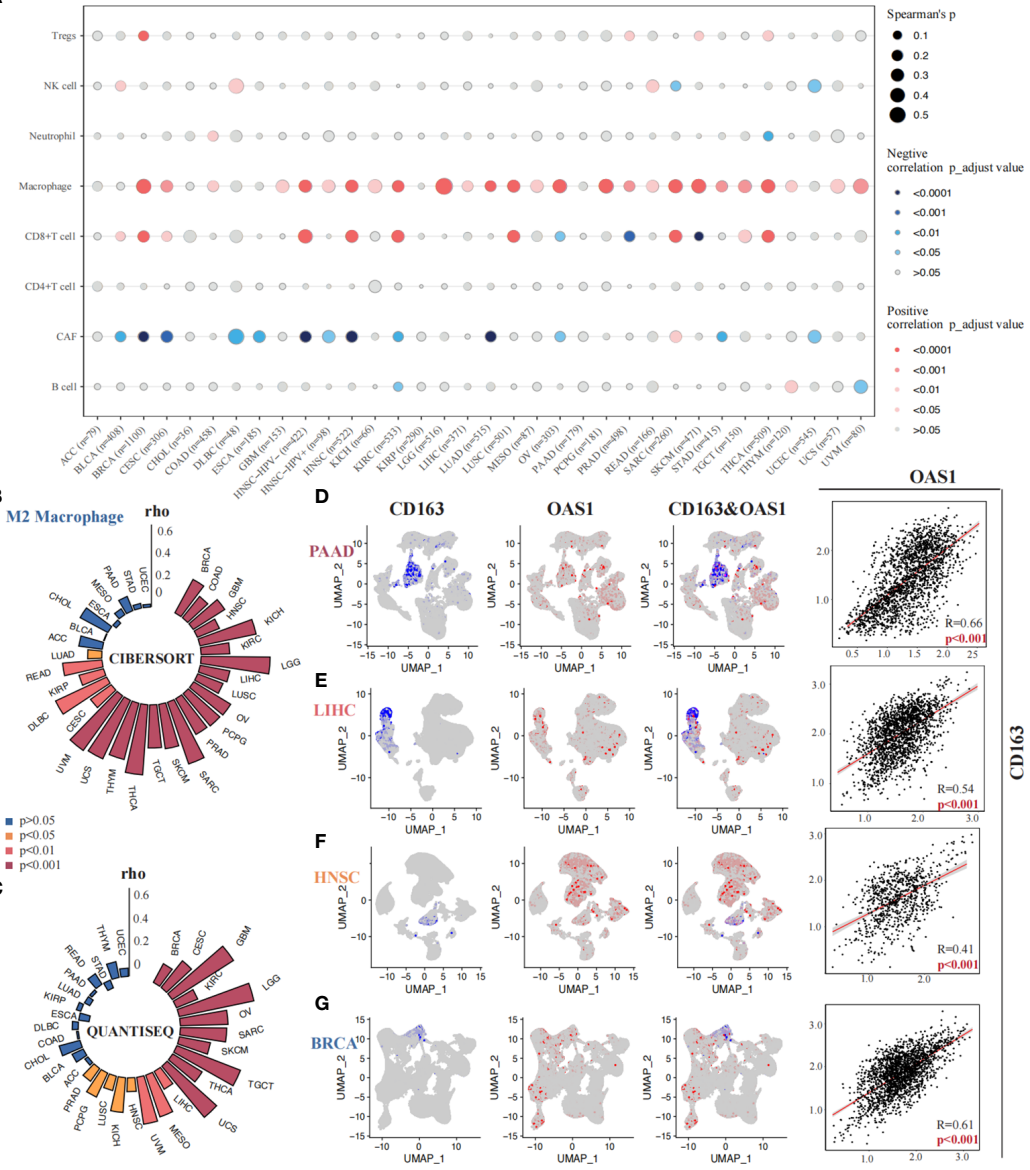
Correlations between OAS1 expression and tumor immune microenvironment. A significant positive correlation between OAS1 expression and macrophage infiltration was observed using the xCell algorithm **(A)**. OAS1 expression and M2 subtype macrophage infiltration was analyzed by the CIBERSORT **(B)** and QUANTISEQ **(C)** algorithms. There was positive correlation between OAS1 expression and CD163 in PAAD (R=0.66, p < 0.001) **(D)**, LIHC (R=0.54, p < 0.001) **(E)**, HNSC (R=0.41, p < 0.001) **(F)**, and BRCA (R=0.61, p < 0.001) **(G)**.

The correlation between OAS1 expression and tumor immune microenvironment was finally investigated across various tumor types. We found that OAS1 expression was positively correlated with tumor immune score, stromal score, and ESTIMATE score in most tumors (**Figures S4A-C**), indicating a significant association with tumor immune infiltration. We further analyzed the relationship between OAS1 expression and specific immune cell types using the xCell algorithm. Results showed a significant positive correlation between OAS1 expression and macrophage infiltration (**Figure 8A**). We also used the QUANTISEQ and CIBERSORT algorithms to analyze the correlation between OAS1 expression and M2 subtype macrophage infiltration. Our findings revealed a positive correlation between OAS1 expression and M2 macrophage infiltration in several tumor types using both algorithms (**Figures 8B, C**). At the single-cell level, we investigated the correlation between OAS1 expression and M2 macrophage biomarkers CD68 and CD163. Our results showed a positive correlation between OAS1 expression and CD163 in PAAD (R=0.66, p < 0.001) (**Figure 8D**), LIHC (R=0.54, p < 0.001) (**Figure 8E**), HNSC (R=0.41, p < 0.001) (**Figure 7F**), and BRCA (R=0.61, p < 0.001) (**Figure 8G**). As expected, OAS1 was also co-expressed with CD68 in these tumors (**Figure S4D**). Finally, we compared the predictive accuracy of OAS1 with that of standard immunotherapy prediction models. We found that OAS1 was more accurate in predicting the efficacy of the Gide2019_PD1_Melanoma cohort (AUC=0.75) compared to the standard immunotherapy prediction model (**Figure S4E**). Our findings suggest that OAS1 could serve as a promising biomarker for predicting tumor immune infiltration and response to immunotherapy, especially M2 macrophage polarization.

### 3.8 Correlations between OAS1 expression and tumor immune microenvironment in PAAD

**Figure 9:**
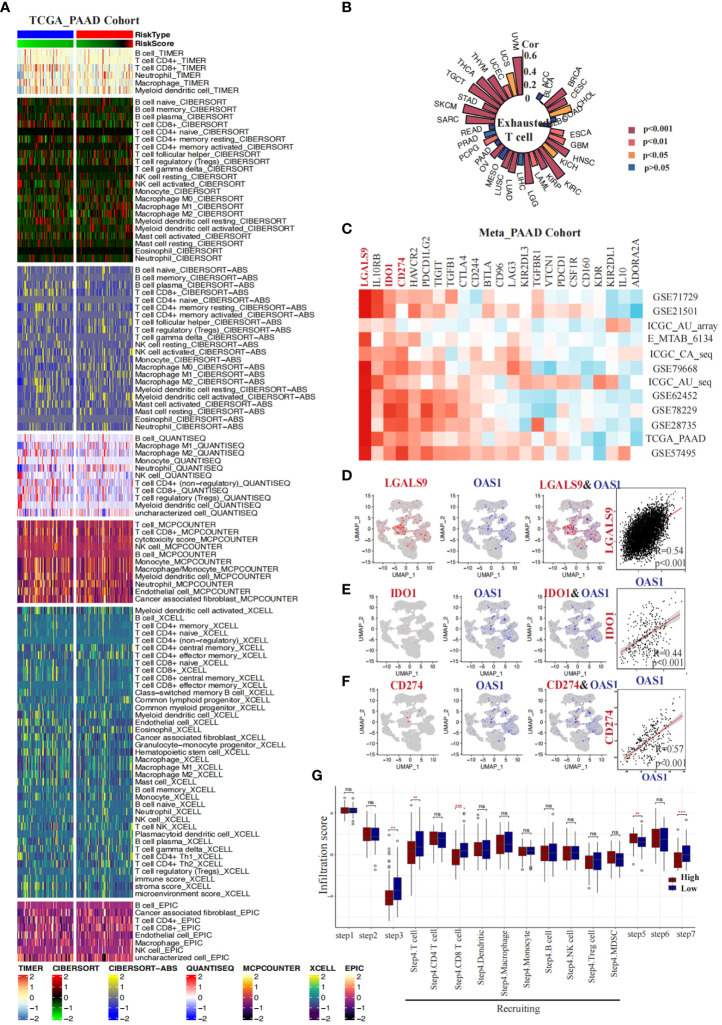
Correlations between OAS1 expression and tumor immune microenvironment in PAAD. Heatmaps display the differences in immune infiltration between the OAS1 high expression group and the OAS1 low expression group using seven algorithms **(A)**. A circular plot illustrates the correlation between OAS1 expression levels and T cell exhaustion scores across different tumor types **(B)**. Heatmaps demonstrate the correlation between OAS1 expression levels and immune checkpoints in various pancreatic cancer datasets **(C)**. At the single-cell level, the correlation between OAS1 expression and LGALS9 **(D)**, IDO1 **(E)**, and CD274 **(F)** is explored. Box plots depict the differences in tumor immune stage scores between the OAS1 high expression group and the OAS1 low expression group **(G)**. #(ns, no significance, **p<0.01, ***p<0.001).

We conducted a comprehensive investigation into the correlation between OAS1 expression and the immune microenvironment in pancreatic cancer. Firstly, we employed seven algorithms (TIMER, QUANTISEQ, CIBERSORT_ABS, MCPCOUNTER, CIBERSORT, XCELL, EPIC) to evaluate the infiltration of distinct immune cells in various samples obtained from the TCGA_PAAD cohort. Subsequently, based on the median value of OAS1 expression, we categorized the samples into two groups: the “OAS1 high expression group” and the “OAS1 low expression group.” We performed an analysis to compare the infiltration of immune cells between these groups (**Figure 9A**). Interestingly, using algorithms such as TIMER (p=0.029), QUANTISEQ (p=0.017), CIBERSORT_ABS (p=0.016), and CIBERSORT (p=0.006), we observed that the group with higher OAS1 expression tended to have lower CD8^+^ T cell scores compared to the group with lower OAS1 expression (**Figures S5A-G**). Furthermore, we delved into the association between OAS1 and exhausted T cells by analyzing the GSCA database. Our investigation revealed a positive correlation between OAS1 expression and T cell exhaustion scores across various tumor types, including BRCA, CESC, HNSC, KIRC, LGG, LUAD, LUSC, OV, SARC, TGCT, THCA, THYM, UVM, *etc.* (**Figure 9B**). In addition, we explored the relationship between OAS1 and immune checkpoints by analyzing multiple pancreatic cancer bulk transcriptome datasets (GSE71729, GSE21501, ICGC_array, E_MTAB_6134, ICGC_CA_seq, GSE79668, GSE62452, GSE78229, GSE28735, TCGA_PAAD, GSE57495). Notably, our findings revealed a positive correlation between OAS1 expression and LGALS9, IDO1, and CD274 in several datasets (**Figure 9C**). Moreover, we extended our investigation to assess the association between OAS1 expression and these three immune checkpoints at the single-cell level, utilizing the PAAD single-cell dataset CRA001160. Intriguingly, at the single-cell level, we observed a positive correlation between OAS1 expression and LGALS9 (R=0.54, p<0.001) (**Figure 9D**), IDO1 (R=0.44, p<0.001) (**Figure 9E**), as well as CD274 (R=0.57, p<0.001) (**Figure 9F**). These compelling results suggest that OAS1 expression is positively linked with the aforementioned immune checkpoints, both at the single-cell and bulk transcriptome levels.Finally, employing the TIP database, we assessed the anticancer immune status across seven distinct stages of the tumor immune cycle, including the release of cancer cell antigens (Step 1), cancer antigen presentation (Step 2), priming and activation (Step 3), trafficking of immune cells to tumors (Step 4), infiltration of immune cells into tumors (Step 5), recognition of cancer cells by T cells (Step 6), and killing of cancer cells (Step 7). Our analysis revealed that the lower OAS1 expression group exhibited a notable increase in the infiltration of CD8+ T cells and displayed enhanced cytotoxic effects on cancer cells, compared to the group with higher OAS1 expression (**Figure 9G**). These findings suggest that reduced OAS1 expression is associated with improved immune cell infiltration and heightened cytotoxicity against cancer cells within the various stages of the tumor immune cycle.

## 4 Discussion

Our study initially provides evidence that OAS1 plays a significant role across multiple tumor types. Notably, OAS1 expression exhibits significant differences between tumor and normal tissue, and it shows significant correlation with tumor staging within BLCA, PAAD, LUAD, SKCM, highlighting its potential as a prognostic marker. In most tumors, the higher the expression of OAS1, the worse the prognosis. We observed that the OAS1 gene can have multiple mutations at different positions in different tumors. Furthermore, the degree of OAS1 copy number variation could indicate tumor prognosis, suggesting potential implications on tumorigenesis and tumor progression. We examined the relationship between OAS1 expression and Tumor Mutational Burden and Microsatellite Instability, both of which have been linked to the formation of tumor neoantigens and the effectiveness of immune checkpoint blockade therapy (64, 65). Despite our findings showing no strong correlation between OAS1 expression and tumor neoantigen formation, with the exception of KIRP, READ, and SKCM, further research is required to elucidate the efficacy of OAS1 expression and immune checkpoint blockade therapy within specific tumors. Interestingly, we also found that OAS1 has a certain correlation with the expression of DNA methyltransferase in some tumors. This is particularly significant as it may offer new insights into epigenetic modifications in tumor progression. Furthermore, we observed that after the OAS1 gene is methylated, the prognosis of cancer patients significantly improves. This may be related to the decrease in expression after OAS1 methylation, which is consistent with our research results in **Figure 2**. This discovery offers novel insights into potential therapeutic interventions, emphasizing the need for further research into the regulatory role of OAS1 in various cancer types. Recent research has shown that OAS1, as a member of the interferon-associated DNA damage resistance signature (IRDS), can enable cancer cells to survive DNA damage by attenuating PAR synthesis and preventing cell death (66). Moreover, IRDS has been found to be a prognostic factor for tumors, with high expression levels promoting tumor progression in oral squamous cell carcinoma and contributing to drug resistance in breast cancer (67–69). Our study demonstrated that knockdown of OAS1 in pancreatic cancer cell lines significantly reduced their invasive ability and increased their apoptosis rate. These findings suggest that OAS1 plays a crucial role in promoting the aggressiveness of cancer.

Previous studies on OAS1 have primarily focused on its role in antiviral and anti-infection mechanisms, highlighting its involvement in the immune system and its correlation with various autoimmune diseases (70). Investigations into the commonalities between autoimmune diseases and cancer have highlighted the crucial role of the IFN-JAK-STAT signal related to STAT1 and OAS1, implying that OAS1 may play a significant role in immune homeostasis in both of these diseases (71). OAS1 is one of the Interferon-Stimulated Genes (ISGs) that are activated by Interferons (IFNs) signaling. Initially, IFNs were considered a type of cytokine with anti-tumor functions, promoting antigen presentation by dendritic cells and activating cytotoxic T cells, and also enhancing the killing of highly immunogenic tumors (72). Nonetheless, growing evidences suggest a clear correlation between the activation of interferon signals and immune checkpoint resistance (73). Prolonged IFNs signals can lead to the acquisition of STAT1-related epigenomic changes (74), which can promote tumorigenesis. Additionally, STAT1 can drive the expression of T-cell inhibitory receptors (75), such as PD-L1, which can bind to the PD-1 on T cells and render them inactive. Not only in tumor immunity, in situations of persistent pathogen infection, continuous activation of IFNs signaling and ISGs can suppress the immune response to avoid excessive immune reactions that may cause damage while keeping the host-pathogen interaction in a state of balance (76). IFN signaling can activate ISGs, and research has shown that ISGs can be prognostic factors for tumors (67). The latest research indicates that the upregulation of OAS1 expression amplifies IFN signaling and sustains a high level of ISGs expression in cancer cells resistant to immune checkpoint blockade (ICB) therapy via the IFN-Inflammatory memory domains (IMDs), consequently augmenting their resistance to immunotherapy (77). OAS1 is a member of the ISG resistance signature (ISG.RS) and has been linked to resistance to immune checkpoint blockade (ICB). Elevated expression levels of OAS1 were observed in ICB-resistant melanoma tumors from Res 499 cell, highlighting its potential role in conferring immunotherapy resistance (78). In our study, we assessed the impact of OAS1 on CTLs. We found that an increased infiltration of CTLs was associated with a worse prognosis in cases where tumors overexpressed OAS1. Previous studies have demonstrated a significant and substantial correlation between the infiltration and functionality of CTLs in the tumor microenvironment and patient prognosis (79). This phenomenon of OAS1 and CTLs could be explained that infiltrating CTLs are exhausted and non-functional in tumors with high expression of OAS1. We found that high expression of OAS1 was associated with the activation of multiple signaling pathways, such as interferon α and γ signaling pathways, as well as the IL6-JAK-STAT3 signaling pathway. As we mentioned earlier, chronic and prolonged INF signal stimulation can induce immunosuppression, facilitating immune evasion in tumors and resistance to immunotherapy. IL6-JAK-STAT3 signaling has also been reported to induce the expression of immune checkpoint molecules (80). Therefore, the high expression of OAS1 in tumor cells is likely to amplify IFN signaling and sustains a high level of ISG expression, then upregulate of immune checkpoint molecules, leading to T-cell exhaustion, immune escape and immunotherapeutic resistance of the tumor.

For more comprehensive insight into the influence of OAS1 within the tumor immune microenvironment, we further analyzed the OAS1 expression and specific immune cell types. OAS1 is primarily expressed in cancer cells and macrophages as a member of the ISG.RS (78). Our findings indicate a strong correlation between OAS1 expression and macrophage infiltration in the tumor microenvironment across various types of cancer, as demonstrated by analyses of both bulk transcriptome data and single cell transcriptome data. Interferon-gamma (IFN-γ), originally identified as the ‘macrophage activating factor,’ can polarize macrophages to M1 type (81). IFN-γ can modulate the activation of human macrophages by targeting the kinases mTORC1 and MNK (82). However, other studies suggest that signals from IFN-I signal plays a significant role in the death of macrophages caused by Mycobacterium tuberculosis infection (83). Gain-of-function variants of OAS1 can induce dysfunction and apoptosis of macrophages in autoimmune disease patients (33). IFN signaling pathways seem to have different effects on macrophages in different situation. Macrophage can be categorized into M1 and M2 phenotypes. Previous studies have shown that tumor-associated macrophages are predominantly of the M2 phenotype, which plays a critical role in promoting tumor growth, invasion, and metastasis (84). Further investigation revealed a positive correlation between the infiltration of M2 macrophages and OAS1 expression. This suggests that OAS1 may also contribute to immune evasion of tumor cells by influencing M2 macrophages. Hitherto, our understanding of the relationship between OAS1 and M2 macrophages remains limited. There is still a significant knowledge gap regarding how OAS1 specifically influences M2 macrophages and their impact on tumor immune evasion and immunotherapeutic resistance. Therefore, more exploration is needed to unravel the precise molecular mechanisms by which OAS1 affects M2 macrophages.

In conclusion, OAS1 serves as a prognostic biomarker with clinical value in the majority of tumors. Our study has provided preliminary insights into the impact of OAS1 on tumor development from a bioinformatics perspective. We emphasize the potential role of OAS1 in tumor immune evasion and immunotherapeutic resistance. Specifically, we propose that OAS1 is consistent association with T cell dysfunction and M2 macrophage infiltration, although the underlying mechanisms require further investigation. Immunotherapy targeting immune checkpoints has been applied in various cancers, but the efficacy is still suboptimal (5). We have observed a correlation between OAS1 and the expression of immune checkpoints, suggesting that OAS1 could be a promising target for intervention. However, it is important to note that our study primarily relies on bioinformatics analysis and lacks experimental validation. Our subsequent studies will incorporate experimental research to enhance our understanding of the role of OAS1 in tumor immunity.

## Data availability statement

The original contributions presented in the study are included in the article/**Supplementary Material**. Further inquiries can be directed to the corresponding authors.

## Ethics statement

All experiments on tumor samples from patients performed were under the supervision of the Ethics Committee of the First Affiliated Hospital of Xi’an Jiaotong University (XITU1AF2022LSK-339). The studies were conducted in accordance with the local legislation and institutional requirements. The participants provided their written informed consent to participate in this study.

## Author contributions

Study concept and design: RY and FN. Acquisition of data: RY, YD, MZ, YL and JX. Analysis and interpretation of data: RY, YD, MZ, RL, BY, JX. Drafting of the manuscript: RY, YD, MZ and HF. Critical revision of the manuscript for important intellectual content: YD, RY, HF, and BY. Obtaining funding: FN, PH and BY. Technical or material support: RL and BY. Supervision: FN and PH. All authors contributed to the article and approved the submitted version.
