# Supplementary material for: Multi-omics analysis reveals interferon-stimulated gene OAS1 as a prognostic and immunological biomarker in pan-cancer
Source: Front Immunol. 2023 Oct 20;14:1249731. doi: 10.3389/fimmu.2023.1249731 (PMC10623006; doi:10.3389/fimmu.2023.1249731)
Supplement: Supplementary file 1 [file DataSheet_1.docx]

Supplementary Material

Multi-omics analysis reveals Interferon-Stimulated Gene OAS1 as a prognostic and immunological biomarker in pan-cancer

Runyu Yang^†^, Yue Du^†^, Mengyao Zhang^†^, Yi Liu, Hui Feng, Ruimin Liu, Bingyu Yang, Jiayi Xiao, Pengcheng He*, Fan Niu*

Department of Hematology, First Affiliated Hospital of Xi’an Jiaotong University, No. 277 Yanta West Road, Xi'an, 710061 Shaanxi, China

*** Correspondence:**Corresponding Author
[hepengcheng@xjtu.edu.cn](mailto:hepengcheng@xjtu.edu.cn); [niufan@xjtufh.edu.cn](mailto:niufan@xjtufh.edu.cn)

† These authors contributed equally to this work and share first authorship

# Supplementary Figures and Tables

## Supplementary Figures


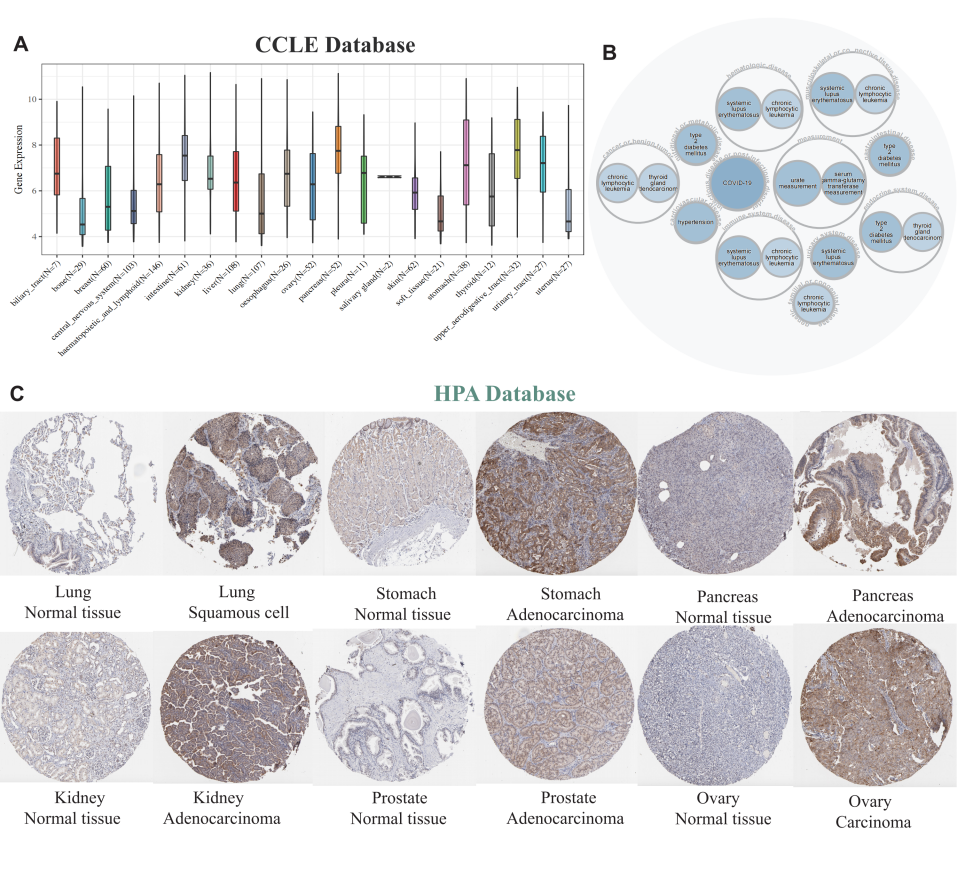


**Supplementary Figure 1.** **The expression of OAS1 in different cancers**

The Cancer Cell Line Encyclopedia (CCLE) database reveals a comparatively elevated expression of the OAS1 gene in pancreatic and intestinal cell lines (A). The OpenTargets database elucidates the connection between the OAS1 gene and diseases such as COVID-19 and Chronic Lymphocytic Leukemia (B). The expression levels of OAS1 in LUAD, STAD, PAAD, KIRC, PRAD, and OV from HPA database (C).


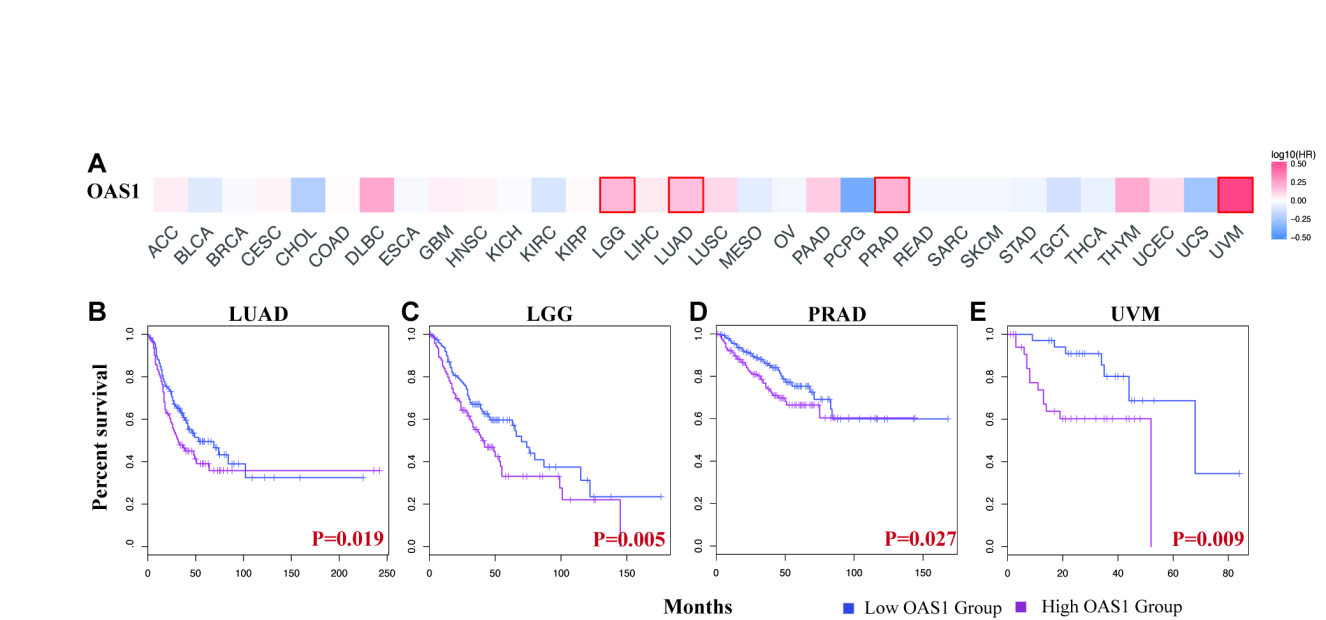


**Supplementary Figure 2. The prognostic value of OAS1 expression**

The expression of OAS1 was significantly overexpressed in LGG, LUAD, PRAD, UVM (A). The higher expression of OAS1 was linked to shorter disease-free survival (DFS) among patients with LUAD (HR=1.4, p =0.019), LGG (HR=1.6，p =0.005), PRAD (HR=1.6, p =0.028), UVM (HR=3.4, p =0.009) (B-E)


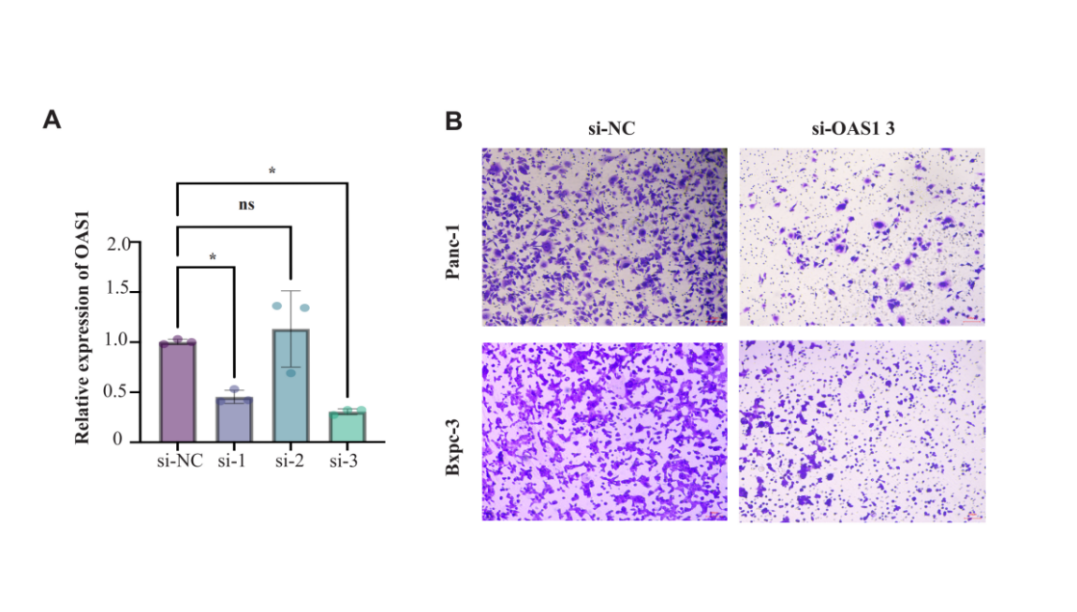


**Supplementary Figure 3. The role of OAS1 in PAAD**

siRNA efficiency was tested by RT-PCR(A). The significant reduction of invasion capability was observed in OAS1 knockdown cell line Bxpc-3 and Panc-1(B). (*p<0.05, **p<0.01, ***p<0.001)


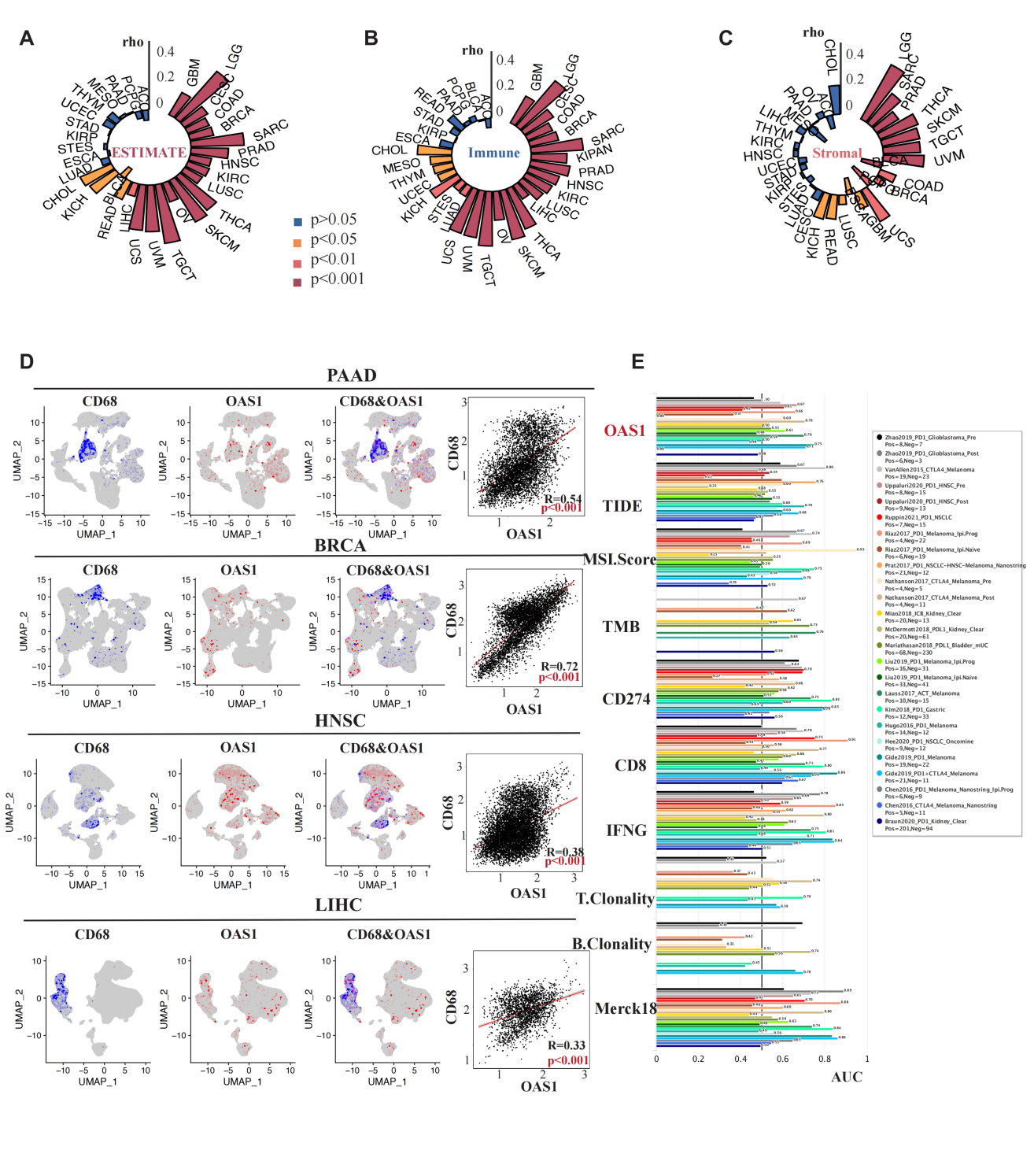


**Supplementary Figure 4. Correlations between OAS1 expression and tumor immune microenvironment**

The OAS1 expression was positively correlated with tumor immune score, stromal score, and ESTIMATE score in most tumors (A-C). OAS1 was co-expressed with CD68 in PAAD, BRCA, HNSC, LIHC (D). OAS1 was more accurate in predicting the efficacy of the Gide2019_PD1_Melanoma cohort (AUC=0.75) compared to the standard immunotherapy prediction model (E).


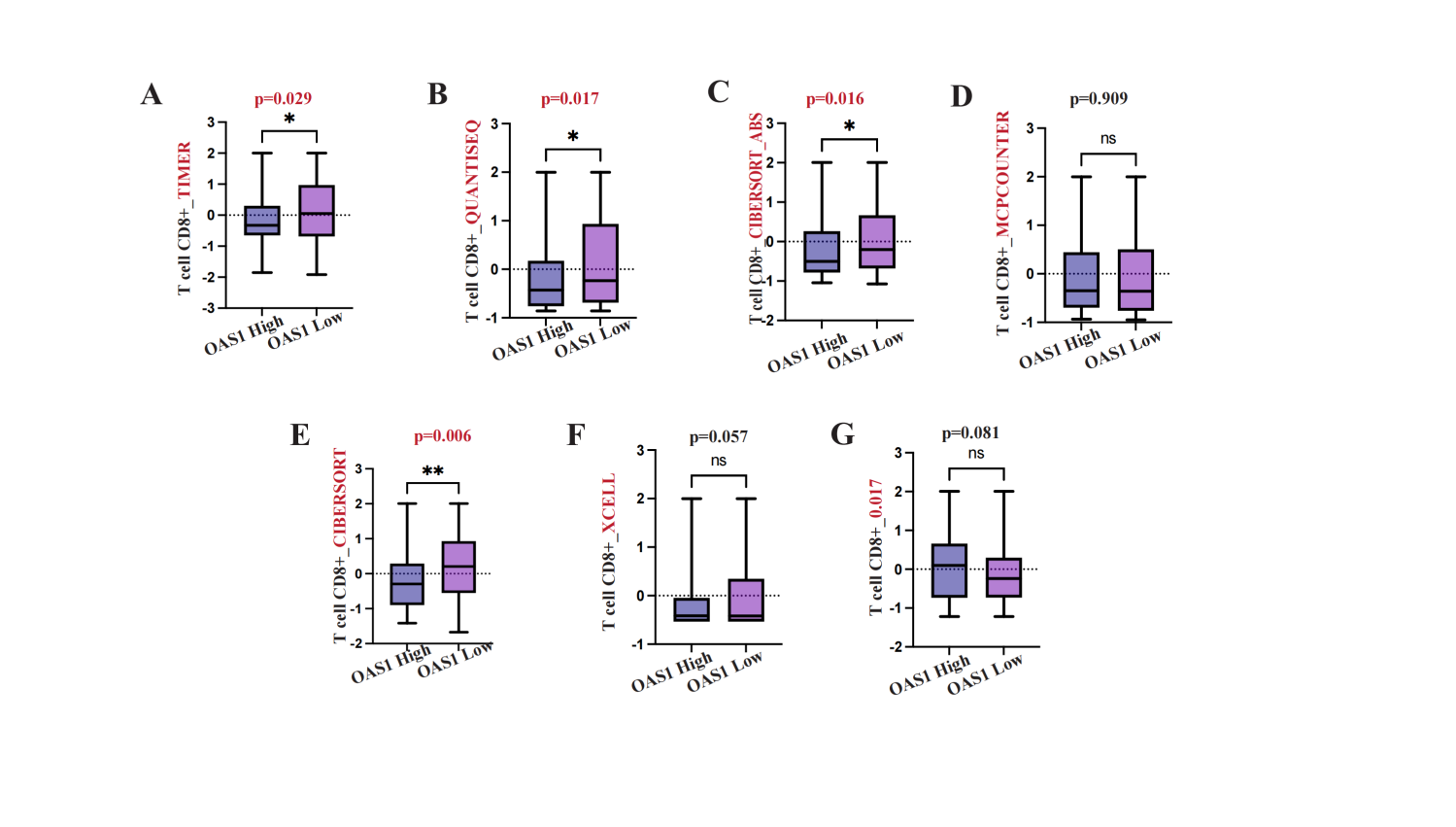


**Supplementary Figure 5. Relationship between OAS1 expression levels and CD8+ T cell levels in PAAD samples.**

The scores of different immune cells in PAAD samples were calculated using seven algorithms, and the differences in CD8^+^ T cell scores between different groups based on OAS1 expression were compared: TIMER(A); QUANTISEQ(B); CIBERSORT_ABS(C); MCPCOUNTER(D); CIBERSORT(E); Xcell(F); EPIC(G).

## Supplementary Tables

**Supplementary Table 1.** SiRNA sequence.

| **Name** |  | **Sequence (5'to3')** |
| --- | --- | --- |
| human-OAS1-siRNA-1-F |  | CAAGUUCAUUGAAGACUAUTT |
| human-OAS1-siRNA-1-R |  | AUAGUCUUCAAUGAACUUGTT |
| human-OAS1-siRNA-2-F |  | GAGUUCAUCCAGGAAAUUATT |
| human-OAS1-siRNA-2-R |  | UAAUUUCCUGGAUGAACUCTT |
| human-OAS1-siRNA-3-F |  | CAUCUACUGGACAAAGUAUTT |
| human-OAS1-siRNA-3-R |  | AUACUUUGUCCAGUAGAUGTT |

**Supplementary Table 2.** Primer sequence.

| **Primer name** | **Primer sequence (5'to3')** |
| --- | --- |
| h-GAPDH-F | CTGGGCTACACTGAGCACC |
| h-GAPDH-R | AAGTGGTCGTTGAGGGCAATG |
| h-OAS1-F | TGACAGGCTGTGCTCCATATT |
| h-OAS1-R | AGGGCTTAGGACAGAACCTGA |

**Supplementary Table 3**. Abbreviations and full names of all TCGA cancers

| **Abbreviation** | **Full name** |
| --- | --- |
| **ACC** | Adrenocortical carcinoma |
| **BLCA** | Bladder urothelial carcinoma |
| **BRCA** | Breast invasive carcinoma |
| **CESC** | Cervical squamous cell carcinoma and endocervi- |
| **CHOL** | Cholangiocarcinoma |
| **COAD** | Colon adenocarcinoma |
| **DLBC** | Lymphoid neoplasm diffuse large B-cell lymphoma |
| **ESCA** | Esophageal carcinoma |
| **GBM** | Glioblastoma multiforme |
| **HNSC** | Head and neck squamous cell carcinoma |
| **KICH** | Kidney chromophobe |
| **KIRC** | Kidney renal clear cell carcinoma |
| **KIRP** | Kidney renal papillary cell carcinoma |
| **LAML** | Acute myeloid leukemia |
| **LGG** | Brain lower grade glioma |
| **LIHC** | Liver hepatocellular carcinoma |
| **LUAD** | Lung adenocarcinoma |
| **LUSC** | Lung squamous cell carcinoma |
| **MESO** | Mesothelioma |
| **OV** | Ovarian serous cystadenocarcinoma |
| **PAAD** | Pancreatic adenocarcinoma |
| **PCPG** | Pheochromocytoma and paraganglioma |
| **PRAD** | Prostate adenocarcinoma |
| **READ** | Rectum adenocarcinoma |
| **SARC** | Sarcoma |
| **SKCM** | Skin cutaneous melanoma |
| **STAD** | Stomach adenocarcinoma |
| **TGCT** | Testicular germ cell tumors |
| **THCA** | Thyroid carcinoma |
| **THYM** | Thymoma |
| **UCEC** | Uterine corpus endometrial carcinoma |
| **UCS** | Uterine carcinosarcoma |
| **UVM** | Uveal melanoma |

**Supplementary Table 4**. The information of Single cell-RNA seq datasets involved in this study.

| **Dataset** | **Sample number** | **Cell number** | **Description** | **Ref.** |
| --- | --- | --- | --- | --- |
| CRA001160 | 35 | 57443 | Single cell-RNA seq data PAAD Patients | 31273297 |
| GSE161529 | 52 | 332168 | Single cell-RNA seq data BRCA Patients | 33950524 |
| GSE172577 | 6 | 57503 | Single cell-RNA seq data HNSC Patients | 34044317 |
| GSE140228 | 5 | 61690 | Single cell-RNA seq data HNSC Patients | 31675496 |

**Supplementary Table 5. The information of bulk transcriptome datasets datasets involved in this study.**

| **Dataset** | **Sample number** | **Description** | **Ref.** |
| --- | --- | --- | --- |
| GSE71729 | 357 | Virtual Microdissection of Pancreatic Ductal Adenocarcinoma Reveals Tumor and Stroma Subtypes | 26343385 |
| GSE21501 | 132 | A six-gene signature predicts survival of patients with localized pancreatic ductal adenocarcinoma | 20644708 |
| GSE79668 | 51 | RNA-sequencing of human pancreatic adenocarcinoma cancer tissues | 27282075 |
| GSE62452 | 130 | Microarray gene-expression profiles of 69 pancreatic tumors and 61 adjacent non-tumor tissue from patients with pancreatic ductal adenocarcinoma | 27197190 |
| GSE78229 | 50 | Microarray gene-expression profiles of 50 pancreatic tumors tissue from patients with pancreatic ductal adenocarcinoma | 27401251 |
| GSE28735 | 45 | Microarray gene-expression profiles of 45 matching pairs of pancreatic tumor and adjacent non-tumor tissues from 45 patients with pancreatic ductal adenocarcinoma | 23918603 |
| GSE57495 | 63 | Microarray analysis of 63 patients with pancreatic cancer tissues resulted in the identification of a 15-gene signature to predict overall survival | 26247463 |
| TCGA-PAAD | 183 | TCGA-GDC | _ |
